# Clinical relevance of St. John's wort drug interactions revisited

**DOI:** 10.1111/bph.14936

**Published:** 2020-01-17

**Authors:** Simon Nicolussi, Jürgen Drewe, Veronika Butterweck, Henriette E. Meyer zu Schwabedissen

**Affiliations:** ^1^ Medical Research Max Zeller Söhne AG Romanshorn Switzerland; ^2^ Biopharmacy, Department of Pharmaceutical Sciences University of Basel Basel Switzerland

## Abstract

The first clinically relevant reports of preparations of St. John's wort (SJW), a herbal medicine with anti‐depressant effects, interacting with other drugs, altering their bioavailability and efficacy, were published about 20 years ago. In 2000, a pharmacokinetic interaction between SJW and cyclosporine caused acute rejection in two heart transplant patients. Since then, subsequent research has shown that SJW altered the pharmacokinetics of drugs such as digoxin, tacrolimus, indinavir, warfarin, alprazolam, simvastatin, or oral contraceptives. These interactions were caused by pregnane‐X‐receptor (PXR) activation. Preparations of SJW are potent activators of PXR and hence inducers of cytochrome P450 enzymes (most importantly CYP3A4) and P‐glycoprotein. The degree of CYP3A4 induction correlates significantly with the hyperforin content in the preparation. Twenty years after the first occurrence of clinically relevant pharmacokinetic drug interactions with SJW, this review revisits the current knowledge of the mechanisms of action and on how pharmacokinetic drug interactions with SJW could be avoided.

**Linked Articles:**

This article is part of a themed section on The Pharmacology of Nutraceuticals. To view the other articles in this section visit http://onlinelibrary.wiley.com/doi/10.1111/bph.v177.6/issuetoc

**Abbreviations**
ABCB1: ATP‐binding cassette subfamily B member 1
CYP: cytochrome P450 enzyme
DSM: diagnostic and statistical manual of mental disorders
EMA: European Medicines Agency
ESCOP: European Scientific Cooperative on Phytotherapy
HAMD: Hamilton rating scale for depression
HMPC: Committee on Herbal Medicinal Products of the EMA
MDR: multidrug resistance
MRP: multidrug resistance‐related protein
OATP: organic‐anion‐transporting polypetide
OCT: organic cation transporter
PCN: pregnenolone 16α‐carbonitrile
P‐gp: P‐glycoprotein (MDR1)
PXRRE: pregnane X response element
SJW: St. John's wort (*Hypericum perforatum*, L.)
SLC: solute carrier
UGT: uridine 5′‐diphospho‐glucuronosyltransferase

## 1 INTRODUCTION

Preparations of St. John's wort (SJW; *Hypericum perforatum* L.; Clusiaceae) enjoy a long history of use in traditional or folk medicine for treating a diverse range of disorders that includes bacterial and viral infections, respiratory impairment, skin wound, peptic ulcers, and inflammation (Nathan, 2001; Robbers & Tyler, 1999; Schwarz & Cupp, 2000). However, the most common reason for using herbal preparations of SJW is to alter mood for relieve of symptoms associated with mild to moderate depressive episodes or major depression respectively (International Classification of Diseases of the WHO, Version 10 F32 F33, DSM‐V). Several clinical trials have demonstrated mood enhancement with an efficacy that is at least comparable to widely prescribed synthetic antidepressants, such as https://www.guidetopharmacology.org/GRAC/LigandDisplayForward?ligandId=203 (Behnke, Jensen, Graubaum, & Gruenwald, 2002; Schrader, 2000), https://www.guidetopharmacology.org/GRAC/LigandDisplayForward?ligandId=4790 (Szegedi, Kohnen, Dienel, & Kieser, 2005), https://www.guidetopharmacology.org/GRAC/LigandDisplayForward?ligandId=4798 (Brenner, Azbel, Madhusoodanan, & Pawlowska, 2000; Gastpar & Zeller, 2005), or https://www.guidetopharmacology.org/GRAC/LigandDisplayForward?ligandId=357 (Philipp, Kohnen, & Hiller, 1999; Woelk, 2000) and superior to placebo (Gastpar, Singer, & Zeller, 2006; Kasper, Anghelescu, Szegedi, Dienel, & Kieser, 2006; Lecrubier, Clerc, Didi, & Kieser, 2002; Schrader, Meier, & Brattström, 1998; Uebelhack, Gruenwald, Graubaum, & Busch, 2004).

**Figure 1:**
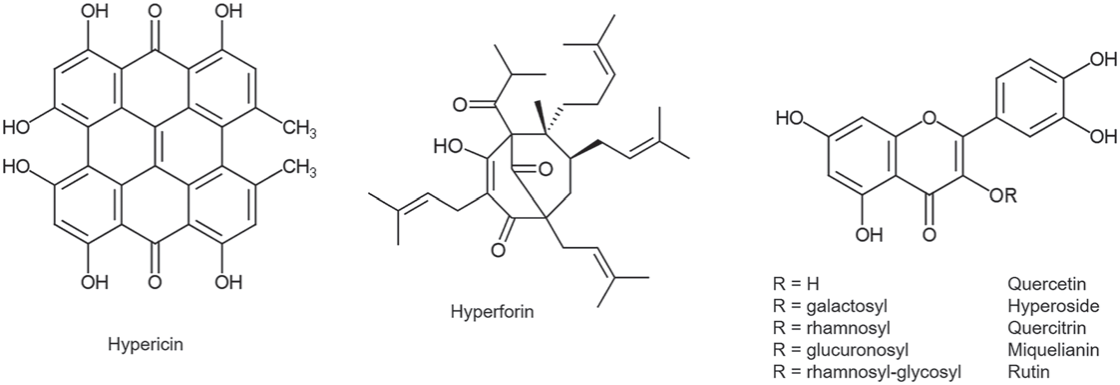
Chemical structures of major compounds found in *Hypericum perforatum* (St. John's wort) extracts

SJW extracts contain numerous constituents belonging to at least 10 biologically active chemical classes (Nahrstedt & Butterweck, 2010). Major compounds are naphthodianthrones (such as hypericin), phloroglucinol derivatives (such as https://www.guidetopharmacology.org/GRAC/LigandDisplayForward?ligandId=2764), and flavonoids (such as https://www.guidetopharmacology.org/GRAC/LigandDisplayForward?ligandId=5346, hyperoside, rutoside, miquelianin, and quercitrin; Figure 1). Numerous SJW preparations are commercially available, and manufacturers employ various methods to produce and maintain uniformity for their products. However, the extraction process determines the composition of the final product. Hydroalcoholic extracts may contain up to 6% hyperforin (Pharm Eur, 01/2017:1874) which is not chemically stable and can degrade rapidly. In the past, the amount of hyperforin was neglected during the extraction process because of its instability, generating hydroalcoholic extracts that usually contained just 0.5–2% hyperforin. However, at the end of the 1990s, some manufacturers modified the extraction method to obtain extracts with hyperforin amounts of 4–5%, because, at that time, hyperforin was thought to be one of the main active compounds in SJW extracts. When the recommended daily dose of SJW is 900 mg (3 × 300 mg) is taken, this amount of the extract is equivalent to a daily dose of approximately 40 mg of hyperforin. Interestingly, along with the modified extraction method producing extracts with a high hyperforin content, first reports of clinically relevant drug interactions occurred. As extracts of natural product, in general, are of complex composition, it is likely that the analytical profile of SJW preparations will vary with the extraction method used. Hyperforin, hypericin, and flavonoids have been demonstrated to be present in very different concentrations in various commercial products. For example, a German study that analysed 33 different SJW products showed that the hyperforin content varied from <0.5 mg per unit (<0.2% of extract) to 13 mg per unit (approx. 4.3% of extract) while hypericin varied between 0.1% and 0.3% (Wurglics et al., 2001a; Wurglics et al., 2001b). Similar results were reported by Länger (2010), who compared the hyperforin and hypericin content of several commercial SJW extracts that were used in relevant clinical studies. The hyperforin content in these extracts varied from 0% to 6%; hypericin varied between 0.1% and 0.3%. In a recent study, Schäfer, Potterat, Seibert, Fertig, & Meyer zu Schwabedissen (2019) tested several commercial SJW extracts currently marketed in Switzerland and observed a clear association between the hyperforin content and the influence on their transactivating activity. Importantly, no such correlation was observed for hypericin content and pregnane X receptor (https://www.guidetopharmacology.org/GRAC/ObjectDisplayForward?objectId=606)‐mediated transactivation.

The general public perception that herbal‐based medicinal products are safe was reinforced by studies showing fewer adverse events occurring with SJW preparations, being possibly even safer than conventional antidepressants (Beaubrun & Gray, 2000; Gaster, 2000; Linde, Berner, & Kriston, 2008; McIntyre, 2000). However, the mood enhancement effect was similar to synthetic antidepressants (Philipp et al., 1999; Schrader, 2000; Schwarz & Cupp, 2000; Woelk, 2000).

A European drug‐monitoring study in 3,250 patients reported an overall adverse events incidence of only 2.4% for the clinical use of a commercial SJW extract in the treatment of depression (Woelk, Brukard, & Grunwald, 1994). Undesirable effects that were most commonly reported were gastrointestinal irritations (0.6%), allergic reactions (0.5%), fatigue (0.4%), and restlessness (0.3%). A meta‐analysis based on traditional SJW preparations revealed that when adverse reactions occur, they are generally mild, transient, and similar to placebo (Linde et al., 1996). In a review of SJW preparations and their adverse drug reactions (ADR), the author noted that this incidence was some 10 times less than that for synthetic antidepressants (Schulz, 2002). The most common adverse events (one per 300,000 treated cases) among the spontaneous reports in a German ADR recording system between October 1991 and December 1999 involved reactions of the skin exposed to light (27 incidents) that was followed by increased bleeding time with coumarin‐type oral anticoagulants (16 reports), eight incidents of breakthrough bleeding with oral contraceptives, and seven reported decreases in https://www.guidetopharmacology.org/GRAC/LigandDisplayForward?ligandId=1024 concentrations in organ transplant recipients. Further investigations in volunteers determined that photosensitisation occurred only when doses of 2–4 g·day^−1^ of a commercial SJW preparation (equivalent to approximately 5–10 mg of the hypericin that causes this effect) were taken (Schulz, 2002). Analysis of available epidemiological data showed that, although photosensitisation had the highest incidence of ADR reports, severe phototoxic reactions comparable to cases documented for grazing animals have never been reported in humans. The 27 phototoxicity reports relative to the incidence of sun exposure damage in the population do not warrant regulatory intervention. The significance of the eight reports of breakthrough bleeding during concomitant SJW and oral contraceptive therapy should be viewed in context with the estimated 4 million female treated patients of child bearing age and the 10‐fold higher incidence of spontaneous breakthrough bleeding with low‐dose oral oestrogens. However, the seven reports of decreased cyclosporine concentrations represent a much higher incidence of ADR in the relatively small transplant patient population (Schulz, 2002). It was discussed at that time that cyclosporine concentrations could indicate dosage adjustment with therapeutic drug monitoring, and in these cases, the interaction would be considered clinically significant.

Since then, several case studies addressed the clinical significance of interactions between cyclosporine and SJW. Awareness of the clinical relevance of this interaction was raised after a liver allograft transplantation in a 63‐year‐old patient. Fourteen months after transplantation, this patient developed severe acute rejection, which was related with an unexpected decrease in cyclosporine levels. The patient had started taking an SJW preparation (2 × 900 mg·day^−1^) for increasing episodes of depression 2 weeks prior to the transplantation. The cyclosporine dosage was then increased, leading to ADRs. Finally, an assessment of oral cyclosporine absorption suggested enhanced cyclosporine metabolism. When SJW intake was discontinued, cyclosporine blood levels recovered (Karliova et al., 2000). Two cases of acute heart transplant rejection that were associated with a specific SJW preparation emphasized the clinical significance of this drug interaction. In both cases, daily dosing with 900 mg of a commercial SJW extract preceded the decreased cyclosporine levels in previously stable patients and acute heart transplant rejection that was demonstrated by endomyocardial biopsy. Cyclosporine concentrations returned to therapeutic range when patients discontinued SJW ingestion (Ruschitzka, Meier, Turina, Lüscher, & Noll, 2000). Nearly identical scenarios were described in two separate case reports for renal transplant patients that had subtherapeutic concentrations of cyclosporine associated with ingesting SJW preparations at recommended doses (Mai et al., 2000; Moschella & Jaber, 2001). In both cases, cyclosporine concentrations returned to normal after discontinuing SJW.

It is noteworthy to mention that for all of the cases where clinical relevant pharmacokinetic interactions occurred, SJW preparations were involved that were rich in hyperforin. For products that contain low‐hyperforin contents, no clinically relevant pharmacokinetic drug interaction has been reported (Table 1; Arold et al., 2005; Mai et al., 2004; Müller et al., 2004; Müller et al., 2006; Müller et al., 2009; Will‐Shahab, Bauer, Kunter, Roots, & Brattström, 2009; Zahner et al., 2019).

**Table 1:** Overview of PK interaction studies with low‐hyperforin SJW preparations

Target enzyme/transporter	Test drug	Hyperforin dose (mg·day^−1^)	Effects on pharmacokinetics	References
CYP 1A2 CYP 2B6 CYP 2C9 CYP 2C19 CYP 2D6 CYP 3A4 P‐gp	Caffeine Bupropion Flurbiprofen Omeprazol Dextromethorphan Midazolam Flurbiprofen	0.96	No clinically relevant interactions.	(Zahner et al., 2019)
CYP 2D6 CYP 3A4	Desogestrel Ethinylestradiol	0.65	No pharmacokinetic interaction with hormonal components.	(Will‐Shahab et al., 2009)
CYP 3A4	Midazolam	0.12	No significant change in C_max_, t_1/2_, t_max_. No clinically relevant interaction.	(Müller et al., 2009)
CYP 3A4	Midazolam	0.13	No clinically relevant interaction.	(Müller et al., 2006)
CYP 1A2 CYP 3A4 P‐gp CYP 2C9	Caffeine Alprazolam Digoxin Tolbutamide	3.5	No significant differences in AUC_0–24_	(Arold et al., 2005)
CYP 3A4 P‐gp	Cyclosporine	0.6	No significant reduction in PK parameters such as AUC_0–12_	(Mai et al., 2004)
P‐gp	Digoxin	0.38	No significant interaction in AUC_0–24_	(Müller et al., 2004)

*Note.* No clinically relevant interactions could be found at indicated low daily doses of hyperforin.

Abbreviations: *C*_max_, maximum plasma concentration; CYP, cytochrome P450 enzyme, P‐gp, P‐glycoprotein; *t*_1/2_, eliminiation *t*_1/2_; *t*_max_, time to reach *C*_max_.

While the use of SJW products in Switzerland, Germany, Austria, and some other European countries is controlled because they are regulated as drugs, SJW preparations are available as dietary supplements in the United States with little regulation and low regulatory hurdles to pass. Based on the Dietary Supplement Health and Education Act of 1994, the U.S. Food and Drug Administration is not authorized to review dietary supplements for safety and effectiveness prior to marketing. While very different with respect to regulatory definition, the terms “dietary supplements” and “herbal supplements” are often used synonymously in the literature. It is beyond the scope of this review to discuss regulatory issues in detail, however, the interested reader is referred to Data S1 of this article where a brief definition of these terms, in view of the associated regulations in various countries is provided.

Twenty years after the appearance of the first reports of clinically relevant drug interactions with SJW, this herbal medicine still attracts significant attention in the matter of safety, efficacy, and mechanism of action. The most important information has been comprehensively summarized (Borrelli & Izzo, 2009; Chrubasik‐Hausmann, Vlachojannis, & McLachlan, 2019; Gurley, Fifer, & Gardner, 2012; Izzo, 2004; Soleymani, Bahramsoltani, Rahimi, & Abdollahi, 2017; Whitten, Myers, Hawrelak, & Wohlmuth, 2006). The present review focuses mainly on the current available knowledge on SJW‐related drug interactions, its clinical efficacy, the possible underlying mechanism of action, and the lessons we have learned from this particular herbal medicine.

## 2 IN VITRO AND IN VIVO PHARMACOLOGICAL MECHANISMS CONTRIBUTING TO THE CLINICAL EFFICACY OF SJW

Up to now, it is impossible to attribute the various pharmacological effects of SJW to the action of single constituents. Therefore, the single compounds of the extract may be regarded to act synergistically (Schmidt & Butterweck, 2015). The extract is considered to be the pharmacological principle, and thus, SJW extracts are classified as *quantified extracts* (Pharm Eur, 01/2017:1874; see also Data S1) by the European regulatory authorities (European Medicines Agency [EMA]/Committee on Herbal Medicinal Products of the EMA [HMPC], 2009).

SJW extracts as well as isolated constituents (hyperforin, hypericin, or flavonoids) have been investigated in vitro and in vivo for their interactions with a variety of potentially relevant targets for depression. However, the current review briefly summarizes only data that were reported for SJW extracts:

- Receptor‐binding studies motivated by the monoamine neurotransmitter hypothesis suggested an interaction with https://www.guidetopharmacology.org/GRAC/DatabaseSearchForward?searchString=serotonin&searchCategories=all&species=none&type=all&comments=includeComments&order=rank&submit=Search+Database, https://www.guidetopharmacology.org/GRAC/DatabaseSearchForward?searchString=dopamine&searchCategories=all&species=none&type=all&comments=includeComments&order=rank&submit=Search+Database, https://www.guidetopharmacology.org/GRAC/ObjectDisplayForward?objectId=408_A_ receptor, https://www.guidetopharmacology.org/GRAC/DatabaseSearchForward?searchString=beta+adrenergic&searchCategories=all&species=none&type=all&comments=includeComments&order=rank&submit=Search+Database, https://www.guidetopharmacology.org/GRAC/DatabaseSearchForward?searchString=corticosteroid&searchCategories=all&species=none&type=all&comments=includeComments&order=rank&submit=Search+Database, https://www.guidetopharmacology.org/GRAC/FamilyDisplayForward?familyId=96, https://www.guidetopharmacology.org/GRAC/FamilyDisplayForward?familyId=2, https://www.guidetopharmacology.org/GRAC/FamilyDisplayForward?familyId=50, and https://www.guidetopharmacology.org/GRAC/FamilyDisplayForward?familyId=75 receptors and https://www.guidetopharmacology.org/GRAC/ObjectDisplayForward?objectId=2489, https://www.guidetopharmacology.org/GRAC/ObjectDisplayForward?objectId=2472, and dopamine hydroxylase (Butterweck, Nahrstedt, et al., 2002; Baureithel, Büter, Engesser, Burkard, & Schaffner, 1997; Cott, 1997; Gobbi, Moia, Pirona, Morazzoni, & Mennini, 2001; Kientsch, Buergi, Ruedeberg, Probst, & Honegger, 2001; Krishnan & Nestler, 2008; Müller & Schäfer, 1996; Rolli, Schäfer, & Müller, 1995; Simmen, Higelin, Berger‐Büter, Schaffner, & Lundstrom, 2001; Wirz et al., 2000; Wonnemann, Schäfer, & Müller, 1997).

The effect of SJW extract on β‐adrenoceptors was first studied by the group of Müller, Rolli, Schäfer, and Hafner (1997) who showed that the number of rat cortical β‐adrenoceptors was down‐regulated after treatment with an SJW extract, while no change in receptor affinity was observed. Kientsch et al. (2001) demonstrated that chronic exposure of an extract, devoid of hyperforin, dose‐dependently down‐regulated the number of β‐adrenoceptors in C6 cells, comparable to desipramine. In vivo, a SJW extract reduced the number of β‐adrenoceptors in rat frontal cortex (Simbrey, Winterhoff, & Butterweck, 2004).

- As observed for https://www.guidetopharmacology.org/GRAC/DatabaseSearchForward?searchString=ssri&searchCategories=all&species=none&type=all&comments=includeComments&order=rank&submit=Search+Database and https://www.guidetopharmacology.org/GRAC/DatabaseSearchForward?searchString=tricyclic+antidepressant&searchCategories=all&species=none&type=all&comments=includeComments&order=rank&submit=Search+Database, re‐uptake inhibition of monoamine neurotransmitters was observed in synaptosomal preparations, brain slices, or neuronal cells (Chatterjee, Bhattacharya, Wonnemann, Singer, & Muller, 1998; Jensen, Hansen, & Nielsen, 2001; Kientsch et al., 2001; Müller et al., 1998; Neary & Bu, 1999; Perovic & Müller, 1995; Ruedeberg, Wiesmann, Brattstroem, & Honegger, 2010; Wonnemann, Singer, Siebert, & Müller, 2001). To explain the mechanism of re‐uptake inhibition, effects of SJW extracts on transporters were investigated. Gobbi et al. (1999) found no interaction of an SJW extract with https://www.guidetopharmacology.org/GRAC/DatabaseSearchForward?searchString=serotonin+transporter&searchCategories=all&species=none&type=all&comments=includeComments&order=rank&submit=Search+Database and explained the re‐uptake inhibitory effects with a reserpine‐like mechanism. Singer, Wonnemann, and Müller (1999) postulated that the re‐uptake inhibition of SJW extract was due to a non‐selective increase in free intracellular sodium concentrations. In vivo, acute and long‐term administration increased brain monoamine neurotransmitter content in the rat cortex after treatment with an SJW extract (Butterweck, Bockers, Korte, Wittkowski, & Winterhoff, 2002).
- Acute immobilization stress following 8 weeks of SJW extract administration decreased mRNA levels of https://www.guidetopharmacology.org/GRAC/DatabaseSearchForward?searchString=serotonin+transporter&searchCategories=all&species=none&type=all&comments=includeComments&order=rank&submit=Search+Database selectively in the rat dentate gyrus (Butterweck, Winterhoff, & Herkenham, 2001). Similar results were observed by Valvassori et al. (2018) who also reported that SJW decreased brain‐derived neurotrophic factors in the rat hippocampus.
- In several neuropsychiatric diseases, including major depression, elevated inflammatory cytokine levels were observed (Miller, Maletic, & Raison, 2009), where microglia seem to be a primary source of brain cytokines. in vitro inhibition of cytokine release was inhibited in PHA/LPS‐stimulated hippocampal HT22 cells (Bonaterra et al., 2018; Thiele, Brink, & Ploch, 1994). An SJW extract also protected rat and human pancreatic islets against cytokine toxicity (Novelli et al., 2014). Furthermore, SJW reduced paracetamol‐induced cytokine production in male Swiss mice (Hohmann et al., 2015).

As a hyperactivity of the hypothalamic–pituitary–adrenal axis appears to be involved in depression (Arborelius, Owens, Plotsky, & Nemeroff, 1999), several investigations showed modulating effects of SJW extracts on this axis (Butterweck, Winterhoff, & Herkenham, 2003; Butterweck et al., 2001). Short‐ and long‐term administration of an SJW extract to rats reduced the expression of genes that are involved in the regulation of the hypothalamic–pituitary–adrenal axis and lowered plasma https://www.guidetopharmacology.org/GRAC/LigandDisplayForward?ligandId=3633 and https://www.guidetopharmacology.org/GRAC/LigandDisplayForward?ligandId=2869 levels.

- Verjee, Weston, Kolb, Kalbhenn‐Aziz, and Butterweck (2018) showed in recent in vitro experiments that the SSRI citalopram as well as a commercial SJW extract could antagonize the dexamethasone stress‐induced increase in expression of the mRNA for FK506‐binding protein 51 (https://www.guidetopharmacology.org/GRAC/DatabaseSearchForward?searchString=fkbp5&searchCategories=all&species=none&type=all&comments=includeComments&order=rank&submit=Search+Database). FKBP5 is a co‐chaperone involved in the translocation of the glucocorticoid receptor (https://www.guidetopharmacology.org/GRAC/ObjectDisplayForward?objectId=625). Activation of GR leads to an up‐regulation of FKBP5 mRNA, which then provides an ultra‐short negative feedback loop for GR sensitivity. *FKBP5* has been shown to play an important role in several mental disorders and stress‐related conditions (Menke, 2019).
- Recently, an SJW extract containing low amounts of hyperforin was investigated for its effect on plasma membrane fluidity in rat C6 glioblastoma cells (Keksel et al., 2019). https://www.guidetopharmacology.org/GRAC/LigandDisplayForward?ligandId=2868, which is increasingly formed under chronic stress conditions, is raising plasma membrane fluidity (Arborelius et al., 1999; Chrousos, 2009). SJW reversed the cortisol‐induced changes completely. In addition, cortisol and the structurally related substance https://www.guidetopharmacology.org/GRAC/LigandDisplayForward?ligandId=2768 were shown to influence the concentration of https://www.guidetopharmacology.org/GRAC/LigandDisplayForward?ligandId=2718 in cellular membranes. These changes in membrane composition and properties affect membrane‐embedded receptors like the β_1_‐adrenoceptor (https://www.guidetopharmacology.org/GRAC/ObjectDisplayForward?objectId=28), leading to slower receptor mobility (Jakobs et al., 2013; Prenner, Sieben, Zeller, Weiser, & Häberlein, 2007). Appropriate to this are findings showing that under repeated SJW extract treatment, a lower activation of β_1_‐adrenoceptors is observed, indicated by a reduced cAMP formation. Therefore, it may be suggested that SJW not only affects the membrane fluidity of neuronal cells but also affects the lateral mobility of membrane‐associated receptors, which subsequently may normalize signal transduction processes in stress‐related diseases such as depression (Keksel et al., 2019).

## 3 BEHAVIOURAL PHARMACOLOGY RELATED TO ANTIDEPRESSANT EFFECTS

The antidepressant effects of SJW extracts have been tested and confirmed in several animal models of depression: Of particular interest is the forced swimming test, in which a good correlation between the decrease of immobility observed and the corresponding clinical potency has been demonstrated (Porsolt, Le Pichon, & Jalfre, 1977). Several studies demonstrated that SJW extracts dose‐dependently decreased immobility time in this model, an effect which was comparable to synthetic antidepressants (Bano & Dawood, 2008; Butterweck, Jurgenliemk, Nahrstedt, & Winterhoff, 2000; Butterweck, Petereit, Winterhoff, & Nahrstedt, 1998; Butterweck, Wall, Lieflander‐Wulf, Winterhoff, & Nahrstedt, 1997; De Vry, Maurel, Schreiber, de Beun, & Jentzsch, 1999; Lozano‐Hernandez et al., 2010; Paulke, Nöldner, Schubert‐Zsilavecz, & Wurglics, 2008; Tian et al., 2014). The tail suspension test, which also measures changes in immobility in rodents after antidepressant treatment, was applied by several investigators (Butterweck, Christoffel, et al., 2003; Machado et al., 2008; Tian et al., 2014) and SJW extracts significantly reduced the time of immobility in this test.

Several studies also demonstrated that various SJW extracts could reduce stress‐induced behavioural deficits in the learned helplessness test (Bhattacharya, Chakrabarti, & Chatterjee, 1998; Chatterjee et al., 1998; Gambarana et al., 1999; Scheggi et al., 2016). The effect on cognition ability was tested in Barnes maze or Morris water maze. SJW alleviated stress and corticosterone related memory impairments (Trofimiuk & Braszko, 2008; Trofimiuk, Holownia, & Braszko, 2011).

In conclusion, a variety of behavioural studies have been performed in animal models and have independently confirmed the antidepressant effects of SJW extracts. Comparing the scientific evidence of clinical efficacy of SJW with the available data on its mechanism of action, it is still unknown how exactly SJW causes its antidepressive effects. A complex multicomponent mixture such as an extract of a medicinal plant does not exert its effects based on one single component. Therefore, over the years, several potential pharmacological targets have been investigated not only with SJW extracts but also with single constituents. Noteworthy, none of the identified single components of SJW extracts has been shown to fully explain the clinical efficacy in the treatment of symptoms of major depressive disorders. Therefore, SJW is a prime example of the entire extract being defined as the active constituent.

## 4 CLINICAL EFFICACY ACHIEVED WITH LOW DAILY DOSES OF SJW EXTRACT OR HYPERFORIN

**Figure 2:**
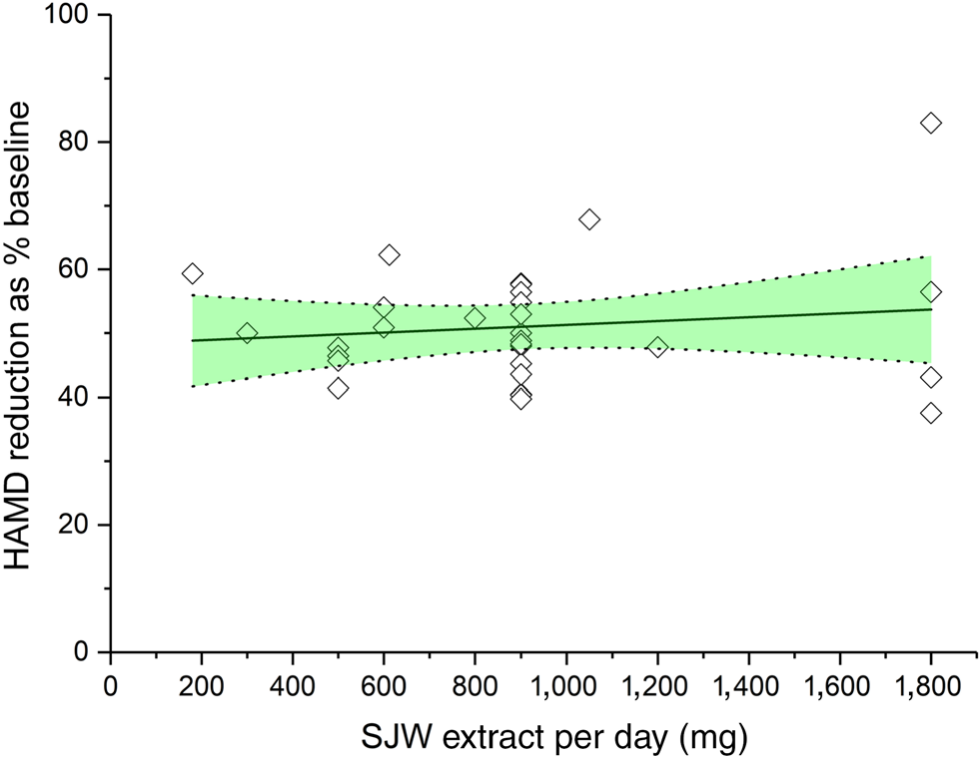
Analysis of clinical efficacy (reduction of HAMD score) induced by different daily doses of SJW extracts. Data are from 30 dose regimens from 28 clinical trials with patients with major depressive disorder. Each data point represents one treatment result at the indicated dosage. No dose‐dependency was found in daily doses ≥180 mg (linear regression slope b = 0.003 ± 0.0042 is not significantly different from 0, *P* = .475, *R* ^2^ = .018), 95% confidence interval is shown in green. No correlation between HAMD reduction and daily dose of SJW was found (Pearson *R* = .136). A saturation effect was observed with doses of 180 mg·day^−1^ and above. Source data as summarized in (EMA/HMPC, 2018). HAMD, Hamilton rating scale for depression; SJW, St. John's wort

The contribution of hyperforin to the clinical efficacy of SJW extracts has been a matter for considerable debate. Initially, the component was suggested as the major active principle of SJW leading to antidepressant effects (Chatterjee et al., 1998). However, clinical efficacy has also been demonstrated with low‐hyperforin SJW extracts (Schrader, 2000; Schrader et al., 1998; Woelk, 2000). When comparing low‐hyperforin (0.5%) versus high‐hyperforin (5%) SJW extracts, no clinically relevant difference could be found (ΔHAMD < minimally clinically important difference of 3 HAMD score points; DGPPN, 2015; Laakmann, Schule, Baghai, & Kieser, 1998). Further, when comparing 600 and 1,200 mg·day^−1^ of a high‐hyperforin SJW extract, no significant difference was observed between the treatment groups (Kasper et al., 2006). When comparing the therapeutic efficacy and daily doses of SJW extracts registered for the treatment of depression, no dose dependency can be found. Rather, a saturation effect at doses ≥180 mg·day^−1^ can be estimated when comparing clinical efficacy data (Figure 2). As summarized in the recent assessment report by the EMA, a broad range of dose regimens were investigated (EMA/HMPC, 2018). Noteworthy, not only do the daily doses of investigated SJW extracts differ widely, from 180 to 1,800 mg, but also their contents of hyperforin vary to an even greater extent, ranging from 0.2% to a maximum of 6%. Such variability has questioned the importance of hyperforin for the clinical efficacy of SJW (Gödtel‐Armbrust, Metzger, Kroll, Kelber, & Wojnowski, 2007; Schäfer et al., 2019; Schmidt & Butterweck, 2015; Wurglics et al., 2001a; Wurglics et al., 2001b). Therefore, in line with its regulatory specification as a “quantified extract,” the whole extract has to be seen as a single active pharmaceutical ingredient contributing to clinical efficacy in the treatment of depressive disorders (Schmidt & Butterweck, 2015).

## 5 LIGAND‐MEDIATED PXR ACTIVATION—THE MECHANISM UNDERLYING THE DRUG‐INTERACTION POTENTIAL OF ST. JOHNS' WORT

It was shortly after the first reports on single cases of SJW being associated with significant changes in pharmacokinetics of the concomitantly used https://www.guidetopharmacology.org/GRAC/ObjectDisplayForward?objectId=1337 substrate cyclosporine (Ahmed, Banner, & Dubrey, 2001; Breidenbach et al., 2000; Ruschitzka et al., 2000), when the underlying mechanism of this marked drug–drug interaction was elucidated. In a well‐designed experimental study, Moore et al. (2000) were able to show that different commercial SJW extracts transactivated the PXR thus activating the transcription and expression of CYP3A4. This enzyme is a member of the enzyme family of https://www.guidetopharmacology.org/GRAC/DatabaseSearchForward?searchString=cytochrome+P450+&searchCategories=all&species=none&type=all&comments=includeComments&order=rank&submit=Search+Database (CYPs) and capable of catalysing oxidative biotransformation reactions (phase I biotransformation). Importantly, CYPs are responsible for the biotransformation of most xenobiotics including more than 50% of all drugs in clinical use (Zanger & Schwab, 2013). Among the 57 putatively functional human CYPs, CYP3A4 but also https://www.guidetopharmacology.org/GRAC/ObjectDisplayForward?objectId=1326, https://www.guidetopharmacology.org/GRAC/ObjectDisplayForward?objectId=1325, https://www.guidetopharmacology.org/GRAC/ObjectDisplayForward?objectId=1330, and https://www.guidetopharmacology.org/GRAC/ObjectDisplayForward?objectId=1319 are most highly expressed in liver, covering a large spectrum of chemical entities handled in metabolism (Zanger & Schwab, 2013). Moreover, Moore et al. (2000) tested several constituents of SJW and showed that hyperforin was the most likely driver of the PXR transactivation, with a *K*_i_ of 27 nM. In the same year, Wentworth, Agostini, Love, Schwabe, and Chatterjee (2000) demonstrated similar results.

Commercial preparations differ significantly in their content of hyperforin, hypericin, and flavonoids. Importantly, the induction of CYP3A4 in intestinal cells correlates with the content of hyperforin, as reported by Gödtel‐Armbrust et al. (2007). Similar results have been recently shown for PXR transactivation, using hepatoma cells for heterologous expression (Schäfer et al., 2019).

The PXR is a member of the family of nuclear receptors and is involved in the regulation of metabolic processes in response to xenobiotics (Pascussi, Gerbal‐Chaloin, Drocourt, Maurel, & Vilarem, 2003). It exhibits a ligand‐binding domain and a DNA‐binding domain and acts as a ligand‐activated transcription factor after heterodimerizing with the https://www.guidetopharmacology.org/GRAC/DatabaseSearchForward?searchString=RXR&searchCategories=all&species=none&type=all&comments=includeComments&order=rank&submit=Search+Database (Evans & Mangelsdorf, 2014; Hyrsova et al., 2019; Moore et al., 2000).

Based on a PXR pharmacophore model developed by Ekins and Erickson (2002), hypericin, the second presumably active ingredient of SJW, would be classified as potential, but non‐potent, activator of the human PXR. However, experimental data show that there is no significant transactivation in cells exposed to hypericin (Moore et al., 2000).

## 6 PXR‐MEDIATED TRANSCRIPTIONAL REGULATION OF A DRUG METABOLIZING GENE NETWORK

**Figure 3:**
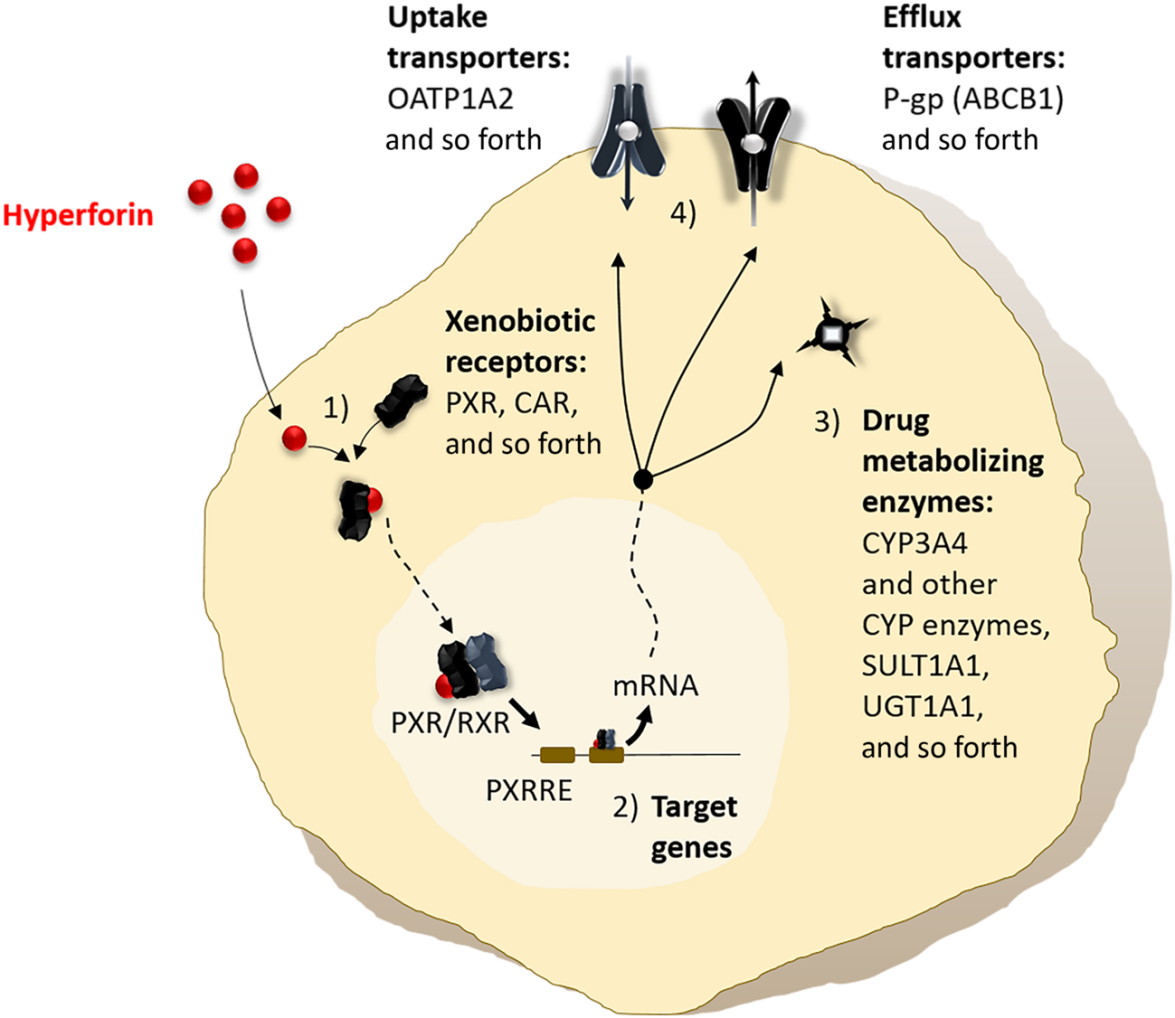
Hyperforin‐dependent mechanism underlies the pharmacokinetic interactions of St. John's wort. ABCB1, ATP‐binding cassette subfamily B member 1; CAR, constitutive active receptor; CYP3A4, cytochrome P450 enzyme 3A4; OATP, organic anion transporting polypetide; PXR, pregnane X receptor; PXRRE, pregnane X response element; RXR, retinoid X receptor; SULT, sulfotransferase

So far, the PXR has evolved into a central regulator of drug metabolism, which not only modulates the activity of CYP3A4, but also of other phase I or phase II metabolizing enzymes, and drug transporters (Tolson & Wang, 2010; Waxman, 1999). In the network of genes involved in drug metabolism, the PXR functions as a xenobiotic receptor or “xenosensor,” which, after ligand binding, translocates to the nucleus, where it binds to specific PXR response elements (PXRRE) in the promotor of various genes, modulating their transcription. Accordingly, this nuclear receptor balances cellular exposure and the activity of the gene network. The function of the gene network is biotransformation and excretion of potentially harmful xenobiotics. The regulation of cytochrome P450 enzymes by PXR is assumed to be one of the mechanisms contributing to the interindividual variability in phase I biotransformation as summarized by Zanger and Schwab (2013). However, considering that drug elimination is based on an interplay of multiple mechanisms, increased clearance can only be achieved if phase I and phase II biotransformation and cellular efflux are modulated at the same time (Figure 3). Testing the influence of in vitro treatment with hyperforin on the mRNA expression in human hepatocytes revealed significantly enhanced expression of https://www.guidetopharmacology.org/GRAC/ObjectDisplayForward?objectId=1324, CYP2C9, CYP3A4, CYP3A5, https://www.guidetopharmacology.org/GRAC/ObjectDisplayForward?objectId=2990, and https://www.guidetopharmacology.org/GRAC/ObjectDisplayForward?objectId=768 (Kandel et al., 2014). For CYP2B6, Goodwin et al. had previously shown the binding of PXR to the promotor. Moreover, they reported increased expression after treatment with known PXR activators including hyperforin (Goodwin, Moore, Stoltz, McKee, & Kliewer, 2001). Chen, Ferguson, Negishi, and Goldstein (2004) showed direct regulation of CYP2C9 by hyperforin‐activated PXR. Finally, UGT1A1 is known to be induced by PXR (Chen, Staudinger, & Klaassen, 2003; Gardner‐Stephen et al., 2004); even if not shown for hyperforin, there is a validated mechanistic link between the nuclear receptor and this enzyme. However, for sulfotransferases, the data on regulation by PXR are less consistent (summarized in Kodama & Negishi, 2013).

## 7 DETAILS ON THE REGULATION OF DRUG TRANSPORTERS BY PXR

Uptake (members of the https://www.guidetopharmacology.org/GRAC/FamilyDisplayForward?familyId=863&familyType=TRANSPORTER family) or efflux transporters (members of the https://www.guidetopharmacology.org/GRAC/FamilyDisplayForward?familyId=136&familyType=TRANSPORTER family) facilitate the transmembrane transport of drugs and are part of the network of genes influencing drug exposure. In terms of regulation by PXR, the transmembrane transporters seem to be modulated differentially. Based on our current knowledge on the transcriptional modulation, there appears to be a more consistent influence of PXR on the protective and/or clearance activity mediated by efflux transporters, than on the cellular or systemic exposure enhancing activity facilitated by uptake transporters. This is certainly true if we are limiting our perspective to the drug uptake transporters: https://www.guidetopharmacology.org/GRAC/FamilyDisplayForward?familyId=238#1220, https://www.guidetopharmacology.org/GRAC/FamilyDisplayForward?familyId=238#1221, https://www.guidetopharmacology.org/GRAC/FamilyDisplayForward?familyId=238#1224, and https://www.guidetopharmacology.org/GRAC/FamilyDisplayForward?familyId=196#1019, which are not directly regulated by PXR. Only for the https://www.guidetopharmacology.org/GRAC/FamilyDisplayForward?familyId=238#1219, are there data suggesting direct modulation of expression in response to PXR activation by https://www.guidetopharmacology.org/GRAC/LigandDisplayForward?ligandId=2765 (Meyer zu Schwabedissen, Tirona, Yip, Ho, & Kim, 2008; Oscarson et al., 2006). This transporter is expressed in the sinusoidal membrane of hepatocytes (Kullak‐Ublick, Stieger, & Meier, 2004), the blood brain barrier (Lee et al., 2005), and other organs. Whether the modulation of OATP1A2 is of functional consequence is currently unknown.

However, for the hepatic uptake transporters OATP1B1 and OATP1B3, the link to PXR‐mediated drug interactions is mostly seen in their influence on the intrahepatocellular accumulation of the ligands (Meyer zu Schwabedissen & Kim, 2009). In the context of the SJW constituents, OATP1B3 is inhibited by hyperforin, suggesting interaction of the compound with this transporter (Smith, Acharya, Desai, Figg, & Sparreboom, 2005). For OATP2B1, we have recently shown that this transporter is not only inhibited by hyperforin but also transports this constituent of SJW (Schäfer, Bock, & Meyer Zu Schwabedissen, 2018), thus influencing the intracellular transactivation of PXR by hyperforin (Schäfer et al., 2019). Importantly, OATP2B1 is expressed not only in human hepatocytes but also in enterocytes (Kobayashi et al., 2003), and cells of the renal tubule (Ferreira et al., 2018), where it is assumed to influence oral drug absorption and renal elimination. Interaction with hyperforin may therefore be not only limited to metabolized substrates but may even be extended.

For the efflux transporters, and especially for ABCB1 (P‐glycoprotein) and https://www.guidetopharmacology.org/GRAC/ObjectDisplayForward?objectId=780 (MRP2), they are known to be transcriptionally regulated by PXR (Geick, Eichelbaum, & Burk, 2001; Kast et al., 2002; Martin, Riley, Back, & Owen, 2008; Oscarson et al., 2006). ABCC2 induction by hyperforin has been reported in human hepatoma cells (Grewal et al., 2017). Similar results were obtained testing the influence of rifampicin in isolated and cultured human hepatocytes (Jigorel, Le Vee, Boursier‐Neyret, Parmentier, & Fardel, 2006; Martin et al., 2008). However, it seems noteworthy that expression and transcriptional functionality of PXR in HepG2 cells is much lower than in isolated human hepatocytes (Martin et al., 2008), suggesting that the observed hyperforin‐associated increase in expression may even be more pronounced. ABCC2 is localized in the canalicular membrane of hepatocytes, where it mediates biliary elimination of various compounds (Konig, Nies, Cui, Leier, & Keppler, 1999). Furthermore, ABCC2 is assumed to be a key determinant in the transmembrane transport of phase II metabolites, although its substrate spectrum is not limited to those metabolites (Fardel, Jigorel, Le Vee, & Payen, 2005). Accordingly, induction of ABCC2 appears to mechanistically and functionally be linked to the enhanced expression and activity of https://www.guidetopharmacology.org/GRAC/FamilyDisplayForward?familyId=988s. A similar mechanistic link is assumed for CYP3A4 and ABCB1 (Kim et al., 1999).

It is assumed that modulation of ABCB1 by PXR is tissue specific, with pronounced changes in enterocytes, but only limited effects in liver in vivo. In detail, Haslam, Jones, Coleman, and Simmons (2008) reported induction of ABCB1 (MDR1) in human intestinal epithelial cells (T84 cells) upon treatment with hyperforin, resulting in significant changes in transepithelial transport of digoxin. In their study, treatment with hyperforin reduced the apical to basal, while enhancing the basal to apical transport of the substrate of ABCB1. In the human colon carcinoma cell line LS147T, Geick et al. (2001) showed a similar effect on ABCB1 expression for rifampicin. The rifampicin‐mediated induction of P‐glycoprotein in enterocytes in vivo had also been shown in an early report by Greiner et al. (1999). However, even if ABCB1 is regulated in human hepatocytes or hepatoma cell lines treated with PXR ligands, there are data suggesting a limited effect on hepatic expression of ABCB1 in patients treated with carbamazepine, suggesting that response to this PXR inducer is tissue specific (Dürr et al., 2000; Oscarson et al., 2006). A similar compartmentalization of the transcriptional response has been observed for the https://www.guidetopharmacology.org/GRAC/ObjectDisplayForward?objectId=607‐inducer efavirenz (Meyer zu Schwabedissen et al., 2012; Oswald et al., 2012).

ABCB1 is a determinant in the protection of the brain, as it functions as a potent efflux pump in the brain capillary endothelial cells, which form the blood–brain barrier. Administration of SJW extract significantly increased the expression of the rodent ABCB1 isoform Mdr1a in the rat hippocampus after 21 days of treatment (Mrozikiewicz et al., 2014), suggesting that there may even be an influence on the functionality of the blood–brain barrier. Bauer et al. also reported induction of P‐glycoprotein expression and function in the blood–brain barrier. Exposing isolated capillaries to PCN (a potent activator of murine PXR) resulted in enhanced expression and function, as shown for the fluorescent cyclosporine derivative (Bauer, Hartz, Fricker, & Miller, 2004). An increase in ABCB1 expression in brain capillaries has also been shown in mice (transgenic for Alzheimer's disease) after 120 days exposure to SJW extracts (Brenn et al., 2014). It is important to mention, in this context, that there is only limited transactivation of the rat PXR by hyperforin, which is significantly enhanced after exchanging the amino acid F305 for leucine (Tirona, Leake, Podust, & Kim, 2004). Accordingly, data reporting on hyperforin effects in rodent models have to be carefully evaluated before being translated. No such species difference has been observed for transactivation of Pxr in cynomolgus monkeys. Indeed, Kim et al. (2010) not only reported a similar EC_50_ for human and cynomolgus Pxr, testing the transactivation by hyperforin in vitro, but were also able to show that SJW (with 0.29 ± 0.02% [*w*/w] hyperforin content) exerts potent induction of midazolam metabolism, an in vivo marker for CYP3A4 activity. Moreover, hyperforin activates the porcine Pxr, thus modulating ABCB1 expression and function in capillaries of pigs (Ott, Fricker, & Bauer, 2009). Moreover, using porcine brain capillary endothelial cells, Ott, Huls, Cornelius, and Fricker (2010) showed that short term exposure to SJW extracts (unknown) or the constituents hyperforin, hypericin, and quercetin (at higher concentrations) inhibited calcein‐efflux function and most likely via ABCB1 (P‐glycoprotein). Finally, using a transgenic mouse model expressing the human isoform of the nuclear receptor, Bauer et al. showed that hyperforin treatment in vitro significantly enhances the expression of ABCB1 (P‐glycoprotein) in brain capillaries. Even if not tested with hyperforin, they were able to show that pretreatment with the PXR‐inducer rifampicin significantly reduced the antinociceptive effect of methadone in mice, even if this treatment did not significantly change the plasma levels of the compound (Bauer et al., 2006). Taken together, it may even be expected that the SJW constituent hyperforin influences the functionality of the blood–brain barrier, thereby enhancing CNS entry of molecules.

## 8 CLINICALLY RELEVANT DRUG INTERACTIONS OF SJW DEPEND ON THE HYPERFORIN DOSE

As mentioned earlier, pharmacokinetic interactions with CYP3A4‐metabolized and/or P‐gp‐transported drugs were reported in cases of acute heart transplant and liver rejection in cyclosporine‐treated patients (Karliova et al., 2000; Ruschitzka et al., 2000) but also in cases of breakthrough bleedings and unwanted pregnancies despite oral contraceptives (Bon, Hartmann, & Kuhn, 1999; Hall et al., 2003; Pfrunder et al., 2003; Schwarz, Buschel, & Kirch, 2003). Further, publications on altered https://www.guidetopharmacology.org/GRAC/LigandDisplayForward?ligandId=4726, https://www.guidetopharmacology.org/GRAC/LigandDisplayForward?ligandId=413, https://www.guidetopharmacology.org/GRAC/LigandDisplayForward?ligandId=6839, and indinavir plasma concentrations were part of the prime safety signals in association with SJW in 1999 (Bon et al., 1999; Cheng, 2000; Johne et al., 1999; Nebel, Schneider, Baker, & Kroll, 1999; Piscitelli, Burstein, Chaitt, Alfaro, & Falloon, 2000). These reports are likely to be the result of the change in extraction procedure triggered by the assumption that hyperforin contributes to the clinical efficacy of SJW (Chatterjee et al., 1998; Madabushi, Frank, Drewelow, Derendorf, & Butterweck, 2006). As a consequence, for SJW products registered as drugs or herbal medicinal products, respective contraindications, warnings, and precautions for use and interactions must be provided in the summary of product characteristics or patient information leaflets (EMA/HMPC, 2009). Related warnings have also to be declared for products in other regulatory categories (Data S1).

Substantiated by the pharmacological mechanism of hyperforin as a PXR‐mediated inducer of metabolic enzymes and transport systems (e.g., CYP450, ABCB1, and OATP1A2), many clinical interaction studies and case reports have been published in causal association with SJW extracts with high‐hyperforin content—(see Chrubasik‐Hausmann et al., 2019; Soleymani et al., 2017) and the current monograph on *Hyperici herba* (European Scientific Cooperative on Phytotherapy [ESCOP], 2018). As concluded by the EMA/HMPC, hyperforin is mainly responsible for pharmacokinetic interactions with other drug substances, which are metabolized by certain CYP450 isoenzymes and transported by ABCB1 (P‐glycoprotein, P‐gp): “The induction of CYP3A4, CYP2C9, CYP2C19 and P‐gp is well documented; the amount is *directly correlated* with the content of hyperforin in the herbal preparation.”

Therefore, with regard to pharmacokinetic interactions, SJW products have to considered in the light of the daily hyperforin dose, leading to a separation of low‐hyperforin SJW preparations (≤1 mg·day^−1^) from high‐hyperforin preparations (>1 mg·day^−1^; EMA/HMPC, 2018; ESCOP, 2018).

No clinically relevant pharmacokinetic interactions have been observed for low‐hyperforin SJW extracts at dosages resulting in up to a maximum dose of 1‐mg hyperforin per day (Table 1). In a recently finalized risk assessment of the EMA, it was stated that adequate studies with extracts with low‐hyperforin content are available which could justify exemptions with regard to contraindications, special warnings, and interactions of the summary of product characteristics (EMA/PRAC, 2018). This statement was provided even before another comprehensive pharmacokinetic interaction study was published, where no clinically relevant interactions were found for seven test drugs in concomitant application with a low‐hyperforin SJW extract (Zahner et al., 2019). As a consequence, convincing clinical evidence prompted the Swiss Agency for Therapeutic Products (Swissmedic) to be the first regulatory authority to approve the removal of contraindications, warnings, and pharmacokinetic interactions for a low‐hyperforin herbal medicinal product.

Taken together, data on the pharmacokinetic interactions with SJW preparations correlate directly with the daily dose of hyperforin (Müller et al., 2006). The induction of PXR‐related metabolic enzymes and transporters cannot be excluded at daily dosages >1‐mg hyperforin. To avoid pharmacokinetic interactions and to contribute to SJW product safety, low‐hyperforin SJW extracts should be recommended for therapeutic use. At daily dosages of maximum 1‐mg hyperforin, no clinically relevant pharmacokinetic interactions are to be expected (EMA/HMPC, 2018; ESCOP, 2018; Zahner et al., 2019).

## 9 CONCLUSIONS

In summary, 20 years after the first reports of clinically relevant drug interactions with SJW extracts, it is clear that, in order to reduce or avoid the risk of pharmacokinetic drug interactions of prescribed medicines with preparations of SJW, the use of quantified extracts with a low‐hyperforin content is recommended. Up to a maximum daily dose of 1‐mg hyperforin, no clinically relevant interactions are to be expected.

On the other hand, to make use of the hyperforin‐dependent induction of PXR‐related metabolic enzymes and transport systems, high‐hyperforin SJW extracts should be further investigated as medications. This could either be for clinical purposes such as treatment of Crigler‐Najjar‐Syndrome type II or in pharmacokinetic interaction studies as inducer of CYP3A4, P‐gp, or UGTs.

Importantly, as recommended by the EMA in 2009, the amount of hyperforin should be declared for medicinal products containing SJW. Unfortunately, even for herbal medicinal products, this safety‐relevant recommendation is rarely followed and is completely neglected in botanicals and food/dietary supplements. Also, in clinical pharmacokinetic studies and case reports, the administered SJW products were often lacking sufficient specifications (herbal drug substance or extract, drug‐extract ratio, solvent), especially regarding the hyperforin content. The ongoing use of high‐hyperforin SJW products among botanicals and food/dietary supplements explains why an unnecessary safety risk for pharmacokinetic drug interactions persists in the public, despite labelling and warnings. This fact highlights also the importance of clinical and analytical comparison studies among different SJW preparations to provide information on hyperforin content of considerably safer products. Therefore, to avoid unnecessary drug safety risks in co‐medication therapy, low‐hyperforin SJW extracts should be prescribed to patients suffering from depressive episodes. Currently, the recommended daily intake of SJW varies between 180 and 1800 mg. As higher doses of SJW do not lead to a more pronounced decrease of depressive symptoms, SJW products with lower extract doses should be preferentially recommended to avoid further safety risks.

In countries with regulations of SJW status other than a registered drug, herbal medicinal product or traditional herbal medicinal product, awareness should increase among physicians regarding hyperforin as dose‐dependent inducer of cytochrome P450 enzymes (e.g., CYP3A4), transporters (e.g., P‐gp, OATP1A2), and other PXR‐related targets.

To avoid the risk of unnecessary pharmacokinetic interactions with SJW, a safety threshold of maximum 1‐mg hyperforin per day is recommended.

### 9.1 Nomenclature of targets and ligands

Key protein targets and ligands in this article are hyperlinked to corresponding entries in http://www.guidetopharmacology.org, the common portal for data from the IUPHAR/BPS Guide to PHARMACOLOGY (Harding et al., 2018) and are permanently archived in the Concise Guide to PHARMACOLOGY 2019/20 (Alexander, Christopoulos et al., 2019; Alexander, Cidlowski et al., 2019; Alexander, Fabbro et al., 2019; Alexander, Kelly et al., 2019; Alexander, Mathie et al., 2019).

## CONFLICT OF INTEREST

S.N., J.D., and V.B. are employees of a manufacturer of an SJW herbal medicinal product. H.M. has no conflict of interest to declare.

## Supporting information

Data S1. Supporting Information
