# Supplementary material for: Clinical relevance of St. John's wort drug interactions revisited
Source: Br J Pharmacol. 2020 Jan 17;177(6):1212–26. doi: 10.1111/bph.14936 (PMC7056460; doi:10.1111/bph.14936)
Supplement: Supplementary file 1 — Data S1. Supporting Information [file BPH-177-1212-s001.pdf]

Supplementary 1

Definitions and Regulatory aspects

Herbal medicines are regulated in different ways depending on the territory. Between 2005 and 2012 a clear trend can be found towards prescription medicines or separate herbal medicines categorisation, while a decrease in member states define herbal medicines as OTC/non-prescription categories or dietary supplements (WHO, 2019).

With regard to health claims or the therapeutic use of herbal preparations a classification based on the regulatory definitions and requirements of scientific evidence in terms of efficacy, safety and quality is depicted in Figure 1.

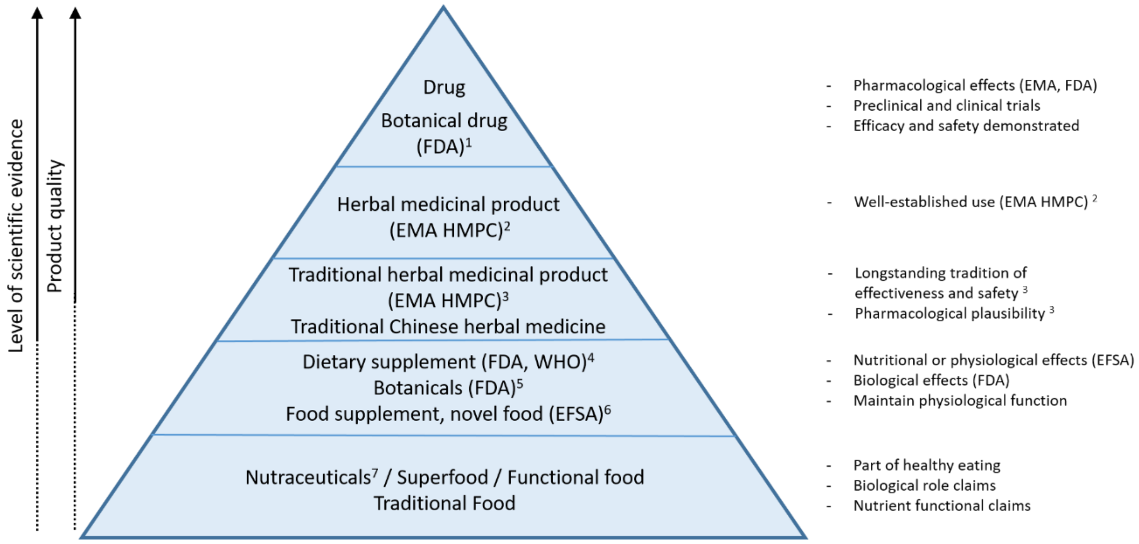

Figure 1: Classification of herbal products with increasing level of scientific evidence. [1](FDA/CDER, 2016), [2]Approved for at least 10 years in the EU with recognised efficacy and acceptable level of safety (Directive_2001/83/EC, 2001). [3] Medicinal use throughout a period of at least 30 years including 15 years in the EU (Directive_2001/83/EC, 2001), [4](FDA, 2015; WHO, 2019), [5](NIH, 2011), [6] *Novel food* is any food that was not used for human consumption to a significant degree within the European Union before May 15th, 1997 (EFSA, 2016; EFSA, 2019), [7](Health_Canada, 2002). EFSA = European Food Safety Authority; EMA = European Medicines Agency; FDA = Food and Drug Administration; HMPC = Committee on Herbal Medicinal Products of the EMA; WHO = World Health Organisation

The figure also demonstrates that there are differences between herbal supplements / herbal medicinal products and dietary supplements, depending on the legal definitions of the regulatory agencies. The correct utilization of definitions needs to be improved notably in the context of safety aspects. Given the fact that self-medication is increasing worldwide, consumers should be aware that dietary supplements although considered as foodstuff can also lead to harmful adverse effects such as drug-interactions. Therefore, the trend towards more specific regulatory classifications of "herbal medicines" is favourable from a medical point of view.

Regulatory categories often including herbal medicines are defined as follows:

- According to the recent WHO global report on traditional and complementary medicine a *dietary supplement* 'could be intended to supplement the diet and will contain, for instance, a vitamin, a mineral, a herb, a botanical or an amino acid. A dietary supplement might also be intended to supplement the diet by increasing the total daily intake of a concentrate, a metabolite, a constituent, an extract or a combination of these ingredients' (WHO, 2019).
- The European Food Safety Authority (EFSA) defines *food supplements* as concentrated sources of nutrients […] or other substances with a nutritional or physiological effect that are marketed in "dose" form […]. A wide range of nutrients and other ingredients might be present in food supplements, including, but not limited to, vitamins, minerals, amino acids, essential fatty acids, fibre and various plants and herbal extracts (EFSA, 2019).
- The FDA defines *dietary supplements* in part as products taken by mouth that contain a dietary ingredient. Dietary ingredients include vitamins, minerals, amino acids, and herbs or botanicals, as well as other substances that can be used to supplement the diet. Further, the FDA suggests to consult a health care professional before using any *dietary supplement*. Many supplements contain ingredients that have strong biological effects, and such products may not be safe in all people. Therefore, appropriate labelling and warning is necessary. Supplements can be harmful when using improperly. Warnings and precautions are providing the consumer with the information, that when using these products with prescribed medicines could lead to harmful, even life-threatening, results (FDA, 2015).
- Botanicals or botanical herbal dietary supplements consist of plants or plant part material valued for medicinal or therapeutic properties. Such preparations are also called herbal products, botanical products of phytomedicines. In the US, such preparations are

to be classified as *dietary supplements*, not to be confused with *botanical drugs* which are intended for use in diagnosing, curing, mitigating, or treating diseases and which are subject to regulation as a drug (FDA/CDER, 2016; NIH, 2011).

- The EMA defines a *herbal medicinal product* as any medicinal product, exclusively containing as active substances one or more herbal substances or one or more herbal preparations, or one or more such herbal substances in combination with one or more such herbal preparations (EMA/HMPC, 2018).
- The EU legal definition differentiates between *herbal medicinal products*, *herbal substances* and *herbal preparations*. *Herbal substances* are ‘whole, fragmented or cut plants, plant parts, algae, fungi, lichen in an unprocessed, usually dried form, but sometimes fresh’. *Herbal preparations* are obtained by extracting, distilling, fractionating, purifying, concentrating or fermenting of herbal substances. Consequently, *herbal medicinal products* contain ‘as active ingredients one or more herbal substances or one or more herbal preparations or herbal substances in combination with one or more herbal preparation’(Directive_2001/83/EC, 2001).

Importantly, an herbal substance or preparation can be categorized either as *standardised*, *quantified* or *other*. *Standardised* herbal substances or preparations are adjusted to a defined content of one or more constituents with known therapeutic activity while *quantified* herbal substances are adjusted to one or more active markers, which are constituents accepted to contribute to a therapeutic activity (EMA/HMPC, 2018).

With regard to herb-drug interactions, the existence of sufficient scientific evidence regarding product safety but also composition (e.g. quantification of responsible constituents) and quality aspects lead to adequate labelling information of a product. In Europe, regulatory classifications of herbal substances provide an important aspect towards product safety (Steinhoff, 2012).

References

Directive_2001/83/EC (2001). ed. Council E.P.a.o.t.

EFSA (2016). Guidance on the preparation and presentation of an application for authorisation of a novel food in the context of Regulation (EU) 2015/2283. EFSA Journal 14.

Food supplements. [Online] Available from https://www.efsa.europa.eu/en/topics/topic/food-supplements. [Accessed: 27.06.2019].

EMA/HMPC (2018). Guideline on quality of herbal medicinal products / traditional herbal medicinal products EMA/HMPC/201116/2005 Rev.3

FDA 101: Dietary Supplements. [Online] Available from https://www.fda.gov/consumers/consumer-updates/fda-101-dietary-supplements. [Accessed: 27.06.2019].

FDA/CDER (2016). Botanical Drug Development - Guidance for Industry, Rev 1.

Nutraceuticals/Functional Foods and Health Claims On Foods. [Online] Available from https://www.efsa.europa.eu/en/topics/topic/food-supplements. [Accessed: 27.06.2019].

Botanical Dietary Supplements. [Online] Available from https://ods.od.nih.gov/factsheets/BotanicalBackground-HealthProfessional/. [Accessed: 27.06.2019].

Steinhoff B (2012). Current perspectives on herb-drug interactions in the European regulatory landscape. Planta Med 78**:** 1416-1420.

WHO (2019). WHO Global Report on traditional and complementary medicine.
